# Optimal experiment selection for parameter estimation in biological differential equation models

**DOI:** 10.1186/1471-2105-13-181

**Published:** 2012-07-27

**Authors:** Mark K Transtrum, Peng Qiu

**Affiliations:** 1 Department of Bioinformatics and Computational Biology, University of Texas M.D. Anderson Cancer Cneter, Houston Texas, USA

**Keywords:** Systems biology, Differential equation models, Experimental design, Parameter estimation, Data fitting

## Abstract

**Background:**

Parameter estimation in biological models is a common yet challenging problem. In this work we explore the problem for gene regulatory networks modeled by differential equations with unknown parameters, such as decay rates, reaction rates, Michaelis-Menten constants, and Hill coefficients. We explore the question to what extent parameters can be efficiently estimated by appropriate experimental selection.

**Results:**

A minimization formulation is used to find the parameter values that best fit the experiment data. When the data is insufficient, the minimization problem often has many local minima that fit the data reasonably well. We show that selecting a new experiment based on the local Fisher Information of one local minimum generates additional data that allows one to successfully discriminate among the many local minima. The parameters can be estimated to high accuracy by iteratively performing minimization and experiment selection. We show that the experiment choices are roughly independent of which local minima is used to calculate the local Fisher Information.

**Conclusions:**

We show that by an appropriate choice of experiments, one can, in principle, efficiently and accurately estimate all the parameters of gene regulatory network. In addition, we demonstrate that appropriate experiment selection can also allow one to restrict model predictions without constraining the parameters using many fewer experiments. We suggest that predicting model behaviors and inferring parameters represent two different approaches to model calibration with different requirements on data and experimental cost.

## Background

A popular class of biological models are differential equations models describing the dynamics of several reactive agents. Models of this type often involve a large number of unknown parameters [1-8] which need to be inferred from experimental data, a process known as model calibration. This inference problem can be very challenging for many reasons. Due to the lack of prior information about parameter values, it is often necessary to search a large region of a high-dimensional space to find parameter values that produce reasonable fits to the data. Furthermore, the models are often highly nonlinear functions of the unknown parameters, making it difficult to navigate this space efficiently. This is particularly true for models which are defined as ordinary differential equations. Challenges notwithstanding, models of this sort have attracted a lot of interest in the systems biology community and much effort has focused on calibrating these models.

Typically, the fitting problem for nonlinear models is very ill-conditioned with large uncertainties in the inferred parameters, a phenomenon sometimes known as *sloppiness*[9-11]. For one model it was observed that inferred parameters had a relative uncertainty of several hundreds of thousands [11], suggesting that inferring parameters accurately might require unreasonable amounts of data. More recent work applying experimental design techniques, however, has shown that parameters for the same model could be inferred with just a few experiments, provided the experiments probed complimentary degrees of freedom [12]. Although the experiments may still require a large amount of data to achieve the desired accuracy [13], they are nevertheless a dramatic improvement over previous results and the present work is motivated in large part by this approach.

More generally, experimental design has been used extensively in guiding modeling of biological systems, see reference [14] for a review. Interest in experimental design has been further motivated by the need to infer topological relationships among biological agents in protein signalling and gene regulatory networks [15]. In general, the relative complexity of models in combination with the limited amount of quantitative data makes optimal experimental design an ongoing challenge in systems biology [12,16-19].

This work is also motivated by the recent 6th Dialogue on Reverse-Engineering Assessment and Methods (DREAM6) parameter estimation challenge [20]. This challenge provided three models and required contestants to “purchase” noisy experimental data on a limited budget with the goal of inferring the model parameters and predicting the time series of the protein concentrations after perturbation. In this work we follow the DREAM6 challenge closely, attempting to infer the parameters and model predictions using the same set of perturbation experiments available in the challenge. However, the DREAM6 challenge was a type of meta-optimization problem; contestants were required to balance the costs of different types of experiments with the goal of estimation accuracy. Although most real-world decisions will hinge on this trade-off, in this study we do not weigh different experiments by their costs. The problem we address is therefore separate from, but related to, that of the DREAM6 challenge. The work described in this paper was conducted after the conclusion of the DREAM6 challenge.

The main result of this paper is that the Fisher Information can be used as an effective criterion for experiment selection. The Fisher Information is a measure of information content based on a local linearization of the model. We show that even when parameter uncertainty is too large to justify the linear approximation, the Fisher Information is still an effective metric for experiment selection. Our method of selecting experiments is therefore computationally efficient since it is based on a sensitivity anslysis at a point estimate of the parameters. It does not require, for example, a sampling of a Bayesian posterior or other rigorous methods of estimating confidence intervals in order to select a maximally informative experiment. It is also robust to which parameter values are used to calculate the Fisher Information. We find that by calculating the Fisher Information at a local minimum rather than the best fit still produces reliable experiment choices to efficiently find the true parameters. Furthermore, our method can be generalized to select experiments that reduce uncertainties in predictions without a need for estimating parameters directly. Indeed, we find that model predictions can often be constrained with considerely less cost than the parameters. In real-world scenarios in which costs must be balanced against research goals, we anticipate this approach to be useful.

In the current approach, we assume that the true mathematical form of the model and the distribution of experimental noise are known, while the model parameters are unknown. Although such assumptions are generally not true in practice, this problem represents a step toward the more general problem of model inference.

## Methods

### Models and data

**Figure 1:**
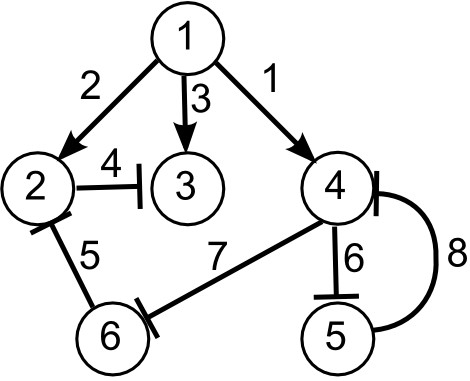
**A graphical representation of model 1.** Six genes are represented by network nodes. The network has eight edges, representing the protein mediated gene interactions.

In this paper, we study three models provided by the recent 6th Dialogue on Reverse-Engineering Assessment and Methods (DREAM6) parameter estimation challenge. These models describe three hypothetical gene-regulatory networks, implemented as ordinary differential equations that describe the time course of 12, 14, and 18 dynamical variables (mRNA and protein concentration for 6, 7, and 9 genes). The goal of the challenge is to select a series of experiments to accurately estimate the model parameters, subject to budgetary constraints. Although our results are valid for all three models, in this presentation we focus on model 1, whose network structure is given in Figure 1. The precise mathematical form of the model is available in several formats from the website of the challenge and given in the appendix.

The unknown model parameters consist of mRNA and protein production and degradation rates, as well as Michaelis-Menten constants and Hill coefficients describing the gene regulation. In our implementation we follow the convention of the DREAM6 challenge and assume that the mRNA degradation rates are each 1, which sets the time scale of the experiments, and that the proteins share a common, albeit unknown, degradation rate. With these conventions, models 1, 2, and 3 have 29, 35, and 49 unknown parameters respectively.

For each model, we generate a set of parameter values and treat them as the true values in our simulation. We then evaluate the time course of the model according to the true parameter values. When evaluating the model, we assume the initial protein concentrations are each 1 while the initial mRNA concentrations are 0. We sample the concentrations for 21 and 41 time points (for mRNA and protein concentrations respectively) evenly spaced between *t* = 0 and *t* = 20, after which, the model has essentially reached the steady state concentration. We then add “experimental” noise to the true time course in the form of both additive and multiplicative Gaussian noise. Specifically, if *v* were the simulated value then the observed value would be given by

(1) $$\;v_{\text{noise}}=\max(0,v+C_{1}\xi_{1}+C_{2}\xi_{2}v),$$

where *ξ*_1_ and *ξ*_2_ are Gaussian random variables with zero mean and standard deviation of one. We use values *C*_1_ = 0.1 and *C*_2_ = 0.2, following the conventions of the DREAM6 challenge.

The noisy time course for mRNA concentrations serve as the startup data, and our goal is to estimate the parameters from this noisy data. However, even knowing the time course for all the dynamical variables of the model is not enough to reasonably constrain the parameters as the parameter can be varied by several orders of magnitude without appreciably changing the model behavior. It is therefore necessary to select new experiments which perturb the model dynamics in order to further constrain the possible parameter values.

### Cost function and minimization scheme

We define a cost function,

(2) $$\;C(\theta )=\frac{1}{2}\underset{i}{\sum }r_{i}{\left(\theta \right)}^{2}$$

where *i* labels each measurements and *θ* are the unknown parameters. The residuals *r*_*i*_ are given by

(3) $$\;r_{i}(\theta )=\frac{y_{i}^{\text{obs}}-y_{i}^{\text{pred}}(\theta )}{\sigma_{i}}.$$

where $y_{i}^{\text{obs}}$ is the *i*-th experimental observation, measured with uncertainty *σ*_*i*_, and $y_{i}^{\text{pred}}(\theta )$ is the corresponding model prediction. The uncertainty is given by $\sigma_{i}=\sqrt{C_{1}^{2}+{\left(C_{2}y_{i}^{\text{obs}}\right)}^{2}}$. Much of the work of this paper involves exploring the dependence of the cost on the unknown parameters *θ*and the choice of experimental data. For a set of data, the parameters _*θBF*_ that globally minimize the cost function in Eq. (2) are known as the best fit.

In practice, even finding a good fit for a large, nonlinear model such as the ones we consider can be a challenging task. When one possesses little or no prior information about expected parameter values, searching a high dimensional parameter space for the optimal fit can be a daunting task. Recent advances have helped to identify the primary pitfalls in finding good fits and suggested methods for finding them more efficiently [21,22].

Algorithms often fail to converge to any minimum of the cost because they push parameter values to their extreme limits (such as zero or infinity), at which point the algorithm fails since the cost function is very flat in these regions. To overcome this problem we follow the method described by Transtrum et al. [22]. First, we augment our cost function with penalties to force the parameters to remain within a reasonable range. These penalties help to guide the algorithm away from extreme parameter values toward ranges where they could be potentially measured by the experiment. Specifically, for each parameter _*θμ*_ we add two additional residuals to our cost function of the form

(4) $$\;r=\sqrt{w_{h}\theta_{\mu }}$$

and

(5) $$\;r=\sqrt{\frac{w_{l}}{\theta_{\mu }}}.$$

The former penalty prevents the parameter _*θμ*_ from becoming too large while the latter prevents it from becoming too small. The weights are chosen to be as small as possible while maintaining a high success rate with the algorithm. We choose _*wh*_ = _*wl*_ = *w*, which places the minimum of the penalty at 1, the natural scale for the problems at hand.

We choose *w* to be 0.1 for the degradation rates and Hill coefficients. This allows the parameters to vary by roughly an order of magnitude in either direction. While this may seem to be is a tight restriction, it is justified in that the models are insensitive to larger variations in these parameters and it would be impossible to estimate them from the data even if the true values were beyond this range. If the final estimate of the true values for the parameters were to lie near the boundaries set by these penalties, they should not be trusted, and a more accurate estimate would require a different set of experimental conditions.

For the remaining parameters we choose *w* = 10^−4^, allowing the parameters to fluctuate by eight orders of magnitude. This larger variation is justified in that the model remains sensitive to these parameters over a larger range. However, as before, if the final estimate of the parameters lies near this boundary, the precise values are suspect.

Under a Bayesian framework, the penalty terms in Eqs. (4) and (5) can be interpreted as priors. However, it is not necessary to adopt a Bayesian approach to justify including the penalties; their practical utility in helping algorithms find the maximum liklihood estimator also makes them useful from a frequentist viewpoint. When performing a frequentist analysis, one would relax the penalities in order to identify the best fit of the bare cost; however, in practice the penalties are weak enough that they make no practical difference in the values of the final parameter estimates. Insetad, their usefulness is in preventing search algorithms from getting lost.

With our modified cost function that includes penalty terms, we search for the best fit parameters using the geodesic Levenberg-Marquardt algorithm [21,22]. The Levenberg-Marquardt algorithm is a gradient search algorithm that interpolates between gradient descent and Newton’s method and is usually the most reliable method for nonlinear least-squares minimization. The geodesic Levenberg-Marquardt attempts to further improve convergence rates by correcting the search direction based on higher order derivative information.

### Error estimation and the fisher information

In the neighborhood of the best fit there exists a region of parameter values that, although not optimal, are nevertheless consistent with the data within experimental noise and constitute the confidence interval for the parameter estimate. The corresponding variation in the parameter values is known as the uncertainty. If it is known that the set of acceptable fits is sufficiently localized around the best fit, then the uncertainty may be estimated by expanding the cost as a Taylor series centered at the minimum:

(6) $$\;C(\theta )\approx C_{0}+\frac{1}{2}\underset{\mu \nu }{\sum }\delta \theta_{\mu }H_{\mu \nu }\delta \theta_{\nu },$$

where the first order terms have vanished since the gradient is zero. The Hessian matrix *H* contains the second derivatives of the cost with respect to the parameters:

(7) $$\begin{array}{ccc} \;H_{\mu \nu } & =\frac{\partial^{2}C}{\partial \theta_{\mu }\partial \theta_{\nu }}\;\\ & =\underset{i}{\sum }\left(\frac{\partial r_{i}}{\partial \theta_{\mu }}\frac{\partial r_{i}}{\partial \theta_{\mu }}+r_{i}\frac{\partial^{2}r_{i}}{\partial \theta_{\mu }\partial \theta_{\nu }}\right)\; & \; \end{array}$$

(8) $$\begin{array}{cc} & \;\approx \underset{i}{\sum }\frac{\partial r_{i}}{\partial \theta_{\mu }}\frac{\partial r_{i}}{\partial \theta_{\mu }}\; \end{array}$$

In the final line we have made the common approximation that the residuals *r*_*i*_ are small near the best fit and can be neglected. This approximate Hessian is the Fisher Information matrix and its inverse is the co-variance of the inferred parameters in the quadratic approximation, which is our approximate parameter uncertainty. In the DREAM6 challenge, the accuracy of contestants’ inferred parameters was measured by the function

(9) $$\;D_{\text{param}}=\frac{1}{N}\underset{i}{\sum }\;{\left[\text{log}\left(\frac{\theta_{i}^{\text{estimate}}}{\theta_{i}^{\text{true}}}\right)\right]}^{2},$$

where *N* is the number of parameters. Because of this, it is advantageous to work in log parameters, essentially measuring relative rather than absolute uncertainty. Additionally, by working in log parameters we enforce that all our parameters are positive, producing an unconstrained optimization problem. In the quadratic approximation, we can estimate our expected value of _*D*param_ as

(10) $$\;D_{\text{param}}\approx \frac{1}{N}\text{trace}\left(I^{-1}\right),$$

where *I* is the Fisher information matrix in log parameters

(11) $$\;I_{\mu \nu }=\underset{i}{\sum }\frac{\partial r_{i}}{\partial \text{log}\theta_{\mu }}\frac{\partial r_{i}}{\partial \text{log}\theta_{\nu }}.$$

Eq. (10) is the average variance of the log parameters, so that a 30% uncertainty in the parameter values corresponds to *D* = 0.3^2^ ≈ 0.1 and a 10% uncertainty corresponds to *D* = 0.1^2^ = 0.01. In practice, the Fisher Information is often ill-conditioned and calculating _*D*param_ from Eq. (10) can often produce numerical errors. Fortunately, in practice, we are primarily interested in the case for which the Fisher Information is the most well-conditioned. We can often disregard the cases in which numerical errors pose problems. The stability of the calculation can further be stabilized by noting that since *I* = *J*^*T*^*J* where $J_{\mathrm{m\mu}}=\partial r_{m}/\partial \;\text{log}\;\theta_{\mu }$, the eigenvalues of *I* are the squares of the singular values of the Jacobian, *J*. In practice, we therefore use $D_{\text{param}}=\underset{\mu }{\sum }1/s_{\mu }^{2}$ where _*sμ*_ are the singular values of the Jacobian. With this approach our calculations do not suffer from numerical instabilities due to the extreme ill-conditionedness of the problem.

Although this approximation of parameter uncertainty is accurate for when the data has sufficiently constrained the parameters, it is not accurate if the uncertainties extend beyond the harmonic approximation or if there are several distinct local minima with reasonable fits. We show, however, that although uncertainties estimated from the Fisher Information may not be accurate, it provides an effective metric to select experiments.

### Experiment selection

With the best fit parameters and an estimate of the uncertainty, we next select an experiment to reduce the estimated error. We consider the same set of potential experiments available in the DREAM6 challenge, which consists of a perturbation to the model and a measurement experiment. The perturbations include deleting one gene, over expressing one of the proteins, or down-regulating the mRNA production of a single gene. The available measurements included the time series of mRNA concentrations (corresponding to a microarray experiment) or of two proteins (corresponding to a fluorescence microscope experiment). We do not consider experiments with different initial conditions, nor do we consider multiple perturbation experiments (i.e. no massive deletions). In addition to time series measurements, we also assume gel-shift assay experiments are available which estimate the true values of the Michaelis-Menten constants and Hill coefficients for a given interaction.

To select an experiment, we simulate all the potential experiments using the current best fit parameters, estimate the parameter error given by Eq. (10) for each experiment, and propose to perform the experiment that reduces the estimated error most. Note that when we evaluate Eq. (10) we do not include the contribution from the penalty terms. In this way Eq. (10) only measure the information content of the experiments. Noisy data corresponding to the selected experiment is then generated by simulating the model with the true parameters and adding noise according to Eq. (1). With the additional data, the previous best fit parameters will no longer lie at a minimum of the cost function. We therefore repeat the process of minimization and error estimation using the new data. We iteratively select experiments in this way until estimated error is sufficiently small.

Our method of selecting experiments is similar to other approaches in the literature [12,19]. The basic scheme is to first estimate the parameters from the available data, either as a point estimate, as we do, or as a Bayesian posterior as done by Vanlier et al. [19]. From this estimate, one predicts the outcome of the available experiments and estimates information content of each experiment. Finally, the most informative experiment is selected and added to the available data and the processes is repeated. We summarize this procedure in Table 1. One of the advantages of our approach is that our method uses a point estimate of the parameters and so does not require a computationally expensive Markov Chain Monte Carlo (MCMC) calculation at each iteration.

**Table 1:** Algorithm for selecting experiments

**Algorithm for selecting experiment**	
1. Find the best fit with available data.	
2. Simulate all possible experiments, assuming the best fit is true.	
3. Evalaute Eq. (10) for each potential experiment.	
4. Select experiment with smallest trace and add the new data to the collection.	
5. Repeat steps 1-4 until parameters are satisfactorily constrained.	

## Results

### Fitting the initial data and estimating uncertainty

By using the methods described above, the fitting process is essentially automated, and we are able to efficiently explore parameter space by searching for local minima of the cost from random initial guesses. With several repeated runs we are able to identify the best fit parameters. There is evidence that the cost landscape for models such as these is very rugged with many local minima [5,23-25]. We therefore also search extensively for local minima.

We explore the extent to which our models have local minima in the cost by searching from 10,000 random starting points chosen uniformly on a log scale in the range corresponding to the penalties described above. The algorithm successfully found a local minimum about 20% of the time for each model. The failures were due to the differential equation solver being unable to accurately integrate the differential equations at extreme values of the parameters. With 10,000 starting points, our sampling of the entire search space is necessarily not very dense, a limitation due to the high-dimensionality of the search space. Notice that the typical number of points per parameter axis for the smallest model is 10000^1/29^ ≈ 1.37 while for the largest model it is 10000^1/49^ ≈ 1.20. However, because such a large fraction of the attempts failed due to extreme parameter values, the sampling is also very diffuse. Therefore, our results cannot be attributed to our search being localized to a small portion of parameter space. Furthermore, this success rate could be increased by reducing the range of starting points. Although the search is sparse and diffuse, if our models had many local minima, we anticipate they would be discovered by our investigation.

Among the roughly 2000 successful attempts of the geodesic Levenberg-Marquardt algorithm, the majority (anywhere from 90% to 99% depending on the model) correspond to “good” fits of the data, i.e. fits within experimental errors. The remaining “bad” fits correspond to fits that fail to fit one or more qualitative features of the data and had much larger values of the cost function than the good fits.

Inspecting the parameter values of the several “good” fits, we find that the parameters vary over a very wide range, suggesting that there are many local minima that fit the data well. However, the eigenvalues structure of the Fisher Information evaluated at these minima suggests that cost surface has many narrow canyons, i.e. there are many very small eigenvalues. Because the cost surface is very flat along the bottom of these canyons, it is possible that these fits actually correspond to the same basin of attraction. Indeed, the cost surface is sufficiently flat along these canyons that one would expect numerical noise, such as rounding errors in the differential equation solver, to create artificial local minima along the bottom of the canyon. We therefore cannot use parameter differences as a criterion for distinguishing distinct local minima.

**Figure 2:**
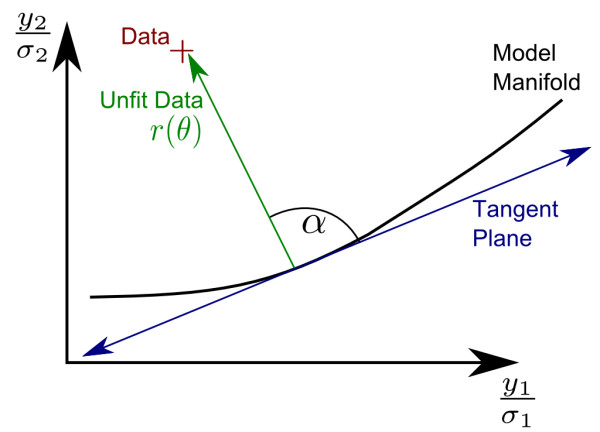
**Convergence criterion for minimization algorithm.** The set of model predictions in data space form a surface known as the model manifold. By comparing the angle between the tangent plane to the *Model Manifold* and the vector of unfit data, we can monitor convergence to a local minimum of the cost. If *α*is approximately a right angle, then we are likely near a local minimum. Fits separated by more than |r(θ)|cosα are likely to correspond to different minima.

A better criterion to identify unique local minima is to use relative differences in the residual vector, _*ri*_(*θ*) as a distinguishing criterion. This is a natural choice as it corresponds to how the search algorithm checks convergence. The algorithm monitors the angle between the unfit residuals and the surface of potential fits in data space, known as the *Model Manifold*[22]. When this angle approaches 90°, the algorithm is near a local minimum as illustrated in Figure 2. Furthermore, from the algorithm’s tolerance we can estimate the distance to the true minimum in data space. If two fits are nearer than the distance specified by the algorithm’s tolerance, then we assume that they belong to the same minimum, even though their parameter values may be very different.

**Figure 3:**
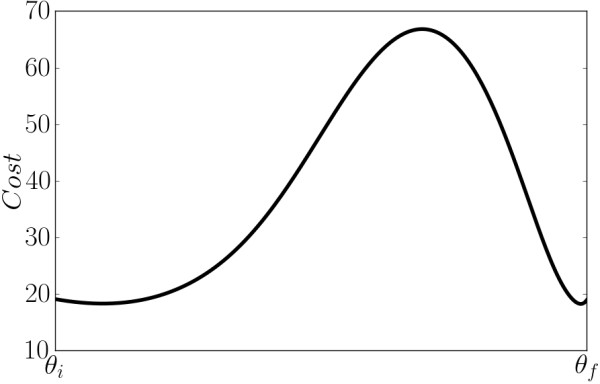
**Cost barrier between two minima.** Although we identified several distinct local minima with that were able to fit the data well, a direct line path connecting these minima reveal that the cost barrier between them is very small. Here we see two points for which the cost barrier between them is about 50. Most randomly selected parameter values have a cost between 1,000 and 10,000, illustrating that this barrier is relatively small. It is possible that these minima are not distinct but are connected by a winding canyon.

Using this criterion, we identify 30 distinct minima for model 1 that were good fits to the data. Closer inspection of the good fits reveals that their residual vectors are separated by a distance only slightly larger than the tolerance specified by the search algorithm. Furthermore, a direct line in parameter space connecting the distinct minima reveals they are separated by very shallow barriers in the cost function as we show in Figure 3. These observations leave open the possibility that these good fits are not actually distinct, but belong to the same basin of attraction. However, it is safe to conclude that even if the good fits are distinct minima, they all reside within some broader basin and are separated only by shallow barriers.

Finally, we compare the orientation of the unconsrained parameter directions as measured by the Fisher Information with a Principal Component Analysis (PCA) of the collection of good fiits. Specifically, consider the two five-dimensional subspaces spanned by the eigenvectors of the least constrained directions for both measures of uncertainty. One can compute the so-called principal angles between these subspaces to measure the extent to which they are aligned [26]. Geometrically, these angles are defined by considering the angles between all the vectors contained in the two subspaces; the smallest such angle is the first principal angle. Smaller angles indicate that the two spaces are more aligned. For the two five-dimensional subspaces we consider, this angle is 16°. By comparison, the average first principal angle among two randomly generated five-dimensional subspaces is approximately 49°(with standard deviation about 6°). From this we conclude that the two subspaces are roughly aligned. Although our local search method produced many different parameter values suggestive of a rough cost surface [5,23-25], our subsequent analysis suggests that these local minima are separated by very small barriers. We therefore believe that the cost functions of our models are dominated by one large basin that is long and narrow, and that the orientation of this basin is roughly described by the Fisher Information (although nonlinearities make the Fisher Information a poor approximation for rigorous confidence intervals). Therefore, by selecting experiments based on the Fisher Information using the criterion in Eq. (10), we hope to maximally increase the curvature of the cost function around this minimum and efficiently estimate the parameter values.

This argument should not be misunderstood to suggest that the linear approximation is an accurate estimate of the uncertainty with sparse data. Rather we expect the Fisher Information to be an efficient choice because of the cost function appears to have only one basin that fits the data well. We can therefore select experiments to maximize the curvature of this basin. This argument will be further strengthened in section Selecting experiments where we show that the choice of experiment is roughly independent of which point in the basin is used to calculate the Fisher Information.

### Selecting experiments

Motivated by the results of the previous section, we assume that experiments that minimize the variance described by the Fisher Information will be maximally informative in constraining the parameters. We therefore select experiments that minimize the error given by Eq. (10) as described in section Experiment selection. An example of experiments produced by this method for model 1 is given in Table 2. This sequence of experiments was generated by finding one local minimum using the startup data, selecting an experiment using the local Fisher information matrix at that minimum, and adding the experimental data for this experiment to the collection of data. We then iteratively repeat the process.

**Table 2:** Experiments for parameter identification in model 1


**Iteration**	**Perturbation**	**Measurement**	**Estimated error**
1	Wild	Microarray	∞
2	Delete 1	Proteins 3 and 4	4.7×10^7^
3	Over 1	Microarray	3.8×10^4^
4	Down 5	Proteins 1 and 6	54
5	Over 1	Proteins 2 and 4	2.5×10^3^
6	Down 5	Microarray	1.5
7	Over 4	Proteins 2 and 4	1.2
8	Down 1	Proteins 2 and 6	1.1
9	Delete 1	Proteins 2 and 6	6.9×10^−2^
10	Assay 1	n/a	2.2×10^−2^
11	Down 5	Proteins 3 and 4	1.5×10^−2^
12	Assay 3	n/a	1.2×10^−2^
13	Down 1	Proteins 3 and 5	1.0×10^−2^

We list the sequence of experiments for estimating the parameters for model 1 for a randomly chosen set of true parameters. Experiments consist of a perturbation and a measurement. “Wild” refers to the original, unperturbed model, while “Delete 1” indicates a deletion of gene 1, “Over 1” indicates an over-expression of gene 1, “Down 5” indicates a down-regulation of protein 5, and so forth. “Microarray” indicates a microarray measurement experiment of time series for all the mRNA concentrations, while “Protein 3 and 4” indicates a time series measurement of proteins 3 and 4, and similarly for the remaining measurements. Gel-shift assay experiments indicate a direct measurement of the Michaelis-Menten constant and Hill coefficients for a specific reaction in the model. The estimated error is calculated from Eq. (10) which is an estimate of the average variance in log-parameters, so that that 30% accuracy corresponds to an error of 0.1 and is achieved after about 9 experiments. An accuracy of 10% corresponds to an estimated error of 0.01 and is achieved after 13 experiments. After about six experiments, the marginal benefit of additional experiments becomes small. At this point, the experiments are no longer complimentary and most of the benefit is attributed to probing the same degrees of freedom with more data. Notice that the estimated error increases at the fifth iteration. This is due to the minimization algorithm not finding the best fit at this iteration. The subsequent success of the method is a demonstration of the robustness of this method for selecting experiments.

**Figure 4:**
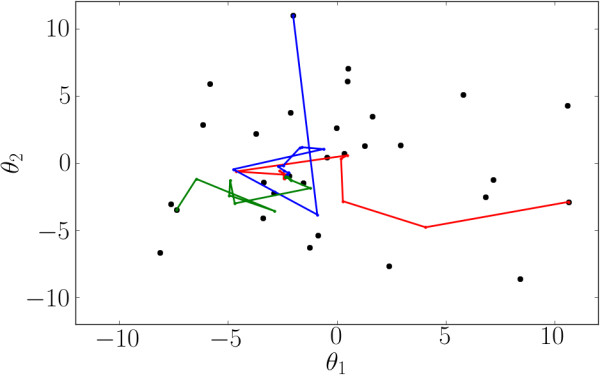
**Paths for distinct minima as data is collected.** The path through parameter space (projected onto the first and second principal component of the ensemble of initial fits) as data is added to minimize the local variance. Although each path corresponds to a different sequence of experiments, they all arrive at the same estimate of the final parameters.

Naturally, the precise sequence of experiments listed in Table 2 depends on at which parameter values the Fisher Information is calculated. However, we repeated the experiment selection procedure from all 30 minima and found that there is much overlap among the selected experiments irrespective of which minimum was used. Additionally, we tested our method using several sets of true parameters and found that the selected experiments are also more or less independent of the true parameter values. Furthermore, after selecting a sequence of experiments, we repeat our search for local minima. We find that the additional data has successfully reduced the 30 minima of the initial startup data into a single good fit, indicating that any of the 30 sequences would have also been effective for estimating the parameters. We see this explicitly in Figure 4 where we show how the experiments guide the paths of the local minima to the true parameter values. Even though the experiments along each path are slightly different, they converge to the same estimate of the true parameters.

It is interesting to compare the uncertainty reduction of our method with that of randomly selected experiments. We find that it typically takes four to five times as many randomly chosen experiments to accomplish a comparable accuracy as that in Table 2.

### Parameters vs predictions

In addition to estimating parameters, the DREAM6 challenge asked contestants to predict a time series of a perturbed version of the model. For model 1 this was a time series for proteins 2, 4, and 6 with several parameters increased by anywhere from a factor of 2 to 10. Contestants were then judged based on their score as measured by

(12) $$\;D_{\text{pred}}=\frac{1}{M}\underset{i}{\sum }\frac{{\left[y_{i}^{\text{pred}}-y_{i}^{\text{true}}\right]}^{2}}{C_{1}^{2}+C_{2}^{2}{\left[y_{i}^{\text{true}}\right]}^{2}},$$

where *M* is the number of predictions. If our goal is to accurately predict the time series, rather than estimating the model parameters, we can modify our criterion of selecting experiments to minimize the expected error as measured by Eq. (12). By extrapolating the uncertainty in the parameters to the uncertainty in the predictions, in the quadratic approximation, the estimate of Eq. (12) becomes

(13) $$\;D_{\text{pred}}=\frac{1}{M}\underset{\mu ,\nu }{\sum }I_{\mu \nu }^{\text{pred}}{\left(I^{-1}\right)}_{\mu \nu },$$

where *I*^pred^ is the Fisher Information for the predicted time series:

(14) $$\;I_{\mu \nu }^{\text{pred}}=\underset{m}{\sum }\frac{\partial y_{m}^{\text{pred}}}{\sigma_{m}\partial \;\text{log}\;\theta_{\mu }}\frac{\partial y_{m}^{\text{pred}}}{\sigma_{m}\partial \;\text{log}\;\theta_{\nu }}.$$

With these modifications, we list a sequence of experiments to minimize the uncertainty in the predictions in Table 3. Notice that only 4 experiments are needed to reduce the uncertainty to within the experimental noise, even with largely unconstrained parameters. If experiments were chosen to infer parameters it would have taken nearly twice as many experiments to get a comparable accuracy.

**Table 3:** Experiments for reducing prediction uncertainty


**Iteration**	**Perturbation**	**Measurement**	**Estimated error**
1	Wild	Microarray	7.7×10^10^
2	Down 5	Proteins 2 and 6	3.5×10^2^
3	Delete 5	Proteins 4 and 6	2.5
4	Over 4	Proteins 4 and 5	2.4×10^−1^

We list the sequence of experiments for estimating the predictions for several protein concentrations in model 1. At each iteration we reduce the relative uncertainty in the predictions given by Eq. (13) which is reported in the estimatd error column. For this measure of error, a value of 1 corresponds to an accuracy comparable to the experimental noise. Notice that the uncertainty in the predictions can be reduced to the experimental uncertainty with just four experiments. Even though the parameters are largely unconstrained after four experiments, the model is nevertheless able to make falsifiable predictions.

## Discussion

Note that the success of the Fisher Information in selecting experiments cannot be attributed to the parameter uncertainty being well-approximated by the linearized residuals. The uncertainties in the inferred parameters after fitting to the startup data are very large and extend well beyond the linear approximation in which the Fisher Information is valid. This can be seen explicitely in Figure 3 in which we show the cost barrier for a straight line path between two good fits. As both endpoints of the path fit the data very well, both points are contained within the confidence region of the parameters. However, since the behavior of the cost along the straight line connecting them is far from quadratic, nonlinearities are clearly important.

**Figure 5:**
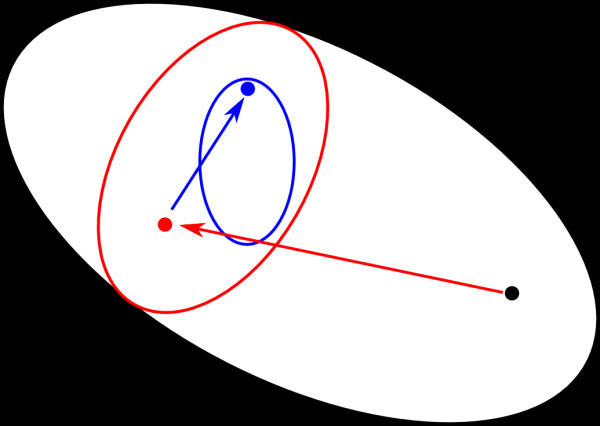
**Confidence interval reduction by experiment selection.** With the initial data, there is a large region of acceptable fits (black) which encompasses all the good fits found in the initial search. Adding a new experiment reduces the confidence region (red) and the best fit moves to a new point within the new confidence region. A second new experiment reduces it further (blue). This process is iterated until the confidence region is acceptably small.

The 30 distinct minima reported in section Fitting the initial data and estimating uncertainty should not be misinterpreted as a sampling of the Bayesian posterior distribution. Rather, they represent potential local minima of the cost surface. As they each fit the data very well, none of these local minima can be ruled out by initial data and additional experiments are necessary to distinguish among them. Although we cannot rigorously identify whether these fits represent local minima or are connected by a flat canyon, by appropriately selecting experiments we were able to successfully distinguish among them. In Figure 5 we interpret the effect of the experiment selection on the confidence interval. The initial confidence region is very large and encompasses all the good fits from the initial search. As additional data are added, the confidence interval shrinks and the location of the minimimum is adjusted. After each additional experiment, the new minimimum lies within the new confidence region.

While the precise order of selected experiments varies depending on the minimum used, there are patterns that can be understood ex post facto by inspecting the network topology. For example, among the list of experiments in Table 2, there is a strong preference for perturbations of gene 1. Inspecting Figure 1 we see that gene 1 acts as a type of head node for the network. It is not regulated by any other gene while any perturbation of gene 1 expression should effect the entire network. It is natural to suspect that experiments perturbing gene 1 will be the most influential. The remaining perturbation experiments involved genes 4 and 5. These experiments can be understood by noting that these genes form a negative feedback loop.

Similarly, we can also understand the choice of measurements. For example, from Figure 1 we see that the protein produced by gene 3 does not regulate any other genes. Consequently, without a measurement of the concentration of protein 3, the parameter controlling the production of protein 3 is completely unconstrained. Because of this, the parameter uncertainty with the initial data is infinite (see Table 2), and we always select a measurement of protein 3 concentrations as the initial experiment.

To understand the remaining measurements, note that there are two channels through which signals are passed, either through genes 1 → 2 → 3 or through genes 1 → 4 → 6. Typically, measurements are selected to observe the effect on both sequences.

The work of Apgar et al. [12] has shown the importance that experiments be selected that contain complimentary information. Our qualitative understanding of the choice of experiments in the previous paragraphs reinforces this claim. Recent work studying the behavior of large nonlinear models, such as these, from an information geometric viewpoint has suggested that models can be interpreted as generalized interpolation schemes [21,22]. We offer an interpretation of the experiment choices from this viewpoint.

**Figure 6:**
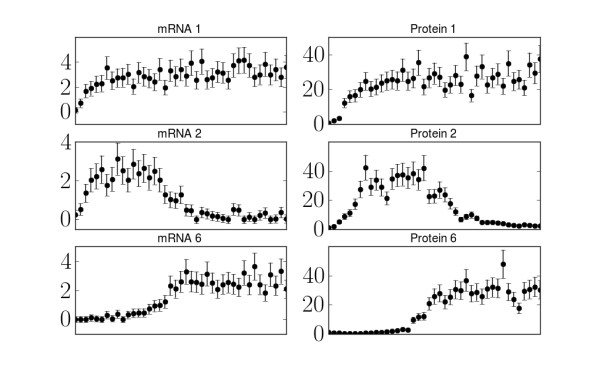
**Independent model degrees of freedom.** Sample data from model 1 for the mRNA and protein concentration corresponding to gene 2 as well as the two genes which regulate these concentrations (genes 1 and 6). By noting that gene 2 is promoted by gene 1 and inhibited by gene 6, it is clear that these time series contain redundant information. The rise and subsequent fall in the mRNA and protein concentrations of gene 2 can be predicted from the time series of genes 1 and 6. Measuring all six time series would be less effective than alternative experiments that probed independent degrees of freedom.

Data leaves parameters unconstrained when it probes fewer effective degrees of freedom than the model has parameters. Consider Figure 6 where we give sample data for model 1. Although the time series corresponding to the concentration of mRNA 1 has many data points, the model effectively has only two degrees of freedom in such a time series: the magnitude of the equilibrium concentration and the time scale it takes to equilibrate. Similarly, the time series of protein 1 has two similar degrees of freedom; however, since protein concentration necessarily echoes its corresponding mRNA concentration, these degrees of freedom are not independent. Furthermore, by noting that gene 2 is promoted by gene 1 and inhibited by gene 6, we see that the six time series in Figure 6 explore many of the same degrees of freedom and are not really independent measurements. In the language of interpolation, the data for gene 2 is, in some sense, “between” the data from genes 1 and 6 and could be inferred by interpolating between the two. Quantitative interpolation is precisely the role of the model. Indeed, even with largely unconstrained parameters, measurements of time series for gene 1 and 6 would be sufficient to give accurate predictions for behavior of gene 2.

The choice of experiments in Table 3 can be understood in a similar manner to those in Table 2. In this case, the three selected experiments are analogous to the perturbations in the desired predictions. Indeed, the primary perturbation in the desired predictions is a massive over-expression of gene 4, consequently, an experiment was selected corresponding to a more mild over-expression of gene 4, as well as experiments to accurately probe the feedback loop that regulates gene 4. The measurement experiments correspond primarily to the three time series to be predicted.

By interpreting the model as a generalized interpolation scheme, we can understand both why the experiments in Table 3 were selected, as well has how the model can make accurate predictions without enough data to constrain the parameters. Just as the time series for gene 2 can be predicted in Figure 6 since its behavior is, in some sense, “between” the behavior of genes 1 and 6, the desired predictions lie “between” the experimental observations in Table 3.

The fact that the cost of inferring parameters is much larger than that of inferring a few predictions (as measured by the relative number of experiments in Tables 2 and 3) suggests two differing approaches to modeling and experimental design. While knowing the parameters has the aesthetic appeal of allowing one to know all potential model predictions, if one is only interested in a few predictions, conducting experiments to infer all the parameters is not a cost effective approach. Furthermore, in most cases it is unknown whether the proposed model represents the true biological network. In these cases, there will likely be several competing models that can only be distinguished by their ability to make falsifiable predictions. The cost advantage of selecting experiments based on the predictions rather than parameter values is likely to be more dramatic.

One could generalize this method of selecting experiments by choosing alternative measures of information. In particular, our criterion in Eq. (10) is based on a linearization of the residuals, and one could construct a more accurate measure for when the linear approximation breaks down. One approach might be based on an MCMC sampling of the Bayesian posterior using Eqs. (4) and (5) as priors or some other appropriate choice. In fact, we have sampled the Bayesian posterior for our models, and find that the allowed range of many parameters is always dominated by the prior for *any* choice of weights. These parameters can fluctuate to infinity or zero with only a statistically insignificant increase in the bare cost. It is therefore a nontrivial problem how to construct a measure of information that adequately reflects the information content of the experiments without being dominated by the prior.

Fortunately, we have shown that Eq. (10) often provides an effective criterion for selecting experiments even when the parameter uncertainties extend well beyond the linear approximation as they do in our case. Indeed, the main result of this paper is that the Fisher Information is efficient for selecting experiments under these conditions. Recall that the Fisher Information describes the curvature of the cost function around a local minimum. Since, this curvature describes the parameter uncertainty in the asymptotic limit, selecting experiments to maximize the curvature is actually a reasonable choice. Experiments which minimize Eq. (10) can be understood as those that bring us closest to the asymptotic regime. We have seen that this argument seems to hold even when the Fisher Information is evaluated at different local minima.

## Conclusion

In this paper we have described a method of selecting experiments to infer unknown model parameters. We have shown that when data is sparse, the parameter uncertainty is large and the cost surface has many local minima. In spite of this, by selecting experiments based on the uncertainty estimated by the local Fisher Information, we are able to reduce parameter uncertainty and constrain the set of reasonable fits to the data to lie within a single region around the best fit. Although this method will produce a different sequence of experiments based upon at which minima the Fisher Information is calculated, we have seen that collection of experiments generated from different minima is in fact very similar.

As we have noted, our method for selecting experiments is very similar to the greedy method described by Apgar et al. [12]. Our work goes beyond previous results, however, in that we have explored the effect of experiment selection on the global cost surface. Since in most cases the parameter uncertainty extends well beyond the harmonic approximation and may even include non-contiguous patches of acceptable parameters around local minima, it is not obvious that a selection criterion that is based only local information (i.e. the Fisher Information) will be effective. Our results help to validate the method of previous work [12], while demonstrating its applicability to additional models.

When selecting experiments, it is important for them to be complimentary and probe independent degrees of freedom of the model. Using the proposition that models should be thought of as a generalized interpolation scheme, we have understood that measurements lying “between” observed data is not as effective at reducing parameter uncertainty as measurements that probe independent degrees of freedom. We have shown that we can qualitatively understand which experiments probe these degrees of freedom by inspecting the network topology of the model.

Previous observations that predictions are often possible without knowing the parameters precisely [2,27] can be understood in this light, as well. Indeed, we have shown that uncertainties in predictions can also be reduced by an adequate series of experiments. Because reducing the uncertainty in a few model predictions does not generally require all the parameters to be tightly constrained, these predictions can be made with fewer experiments.

## Appendix

### Differential equations

In this appendix we give the mathematical form for the first model of the DREAM6 challenge described in this paper.

(15) $$\begin{array}{ccc} \;\frac{d}{\text{dt}}[\text{mRNA}1] & \;= & \text{cod}1-\text{mRNA}\text{\_}\text{deg}\text{\_}\text{rate}\;[\text{mRNA}1]\; \end{array}$$

(16) $$\;\begin{array}{cccc} \;\frac{d}{\text{dt}}[p1] & \;= & \;\text{rbs}1\text{\_}\text{strength} & \;[\text{mRNA}1]\\ & & \;-p\text{\_}\text{deg}\text{\_}\text{rate} & \;[p1] \end{array}$$

(17) $$\begin{array}{ccc} \;\frac{d}{\text{dt}}[\text{mRNA}2] & \;= & \text{cod}2-\text{mRNA}\text{\_}\text{deg}\text{\_}\text{rate}\;[\text{mRNA}2] \end{array}$$

(18) $$\;\begin{array}{cccc} \;\frac{d}{\text{dt}}[p2] & \;= & \;\text{rbs}2\text{\_}\text{strength} & \;[\text{mRNA}2]\\ & & \;-p\text{\_}\text{deg}\text{\_}\text{rate} & \;[p2] \end{array}$$

(19) $$\begin{array}{ccc} \;\frac{d}{\text{dt}}[\text{mRNA}3] & \;= & \text{cod}3-\text{mRNA}\text{\_}\text{deg}\text{\_}\text{rate}\;[\text{mRNA}3]\; \end{array}$$

(20) $$\;\begin{array}{cccc} \;\frac{d}{\text{dt}}[p3] & \;= & \;\text{rbs}3\text{\_}\text{strength} & \;[\text{mRNA}3]\\ & & \;-p\text{\_}\text{deg}\text{\_}\text{rate} & \;[p3] \end{array}$$

(21) $$\begin{array}{ccc} \;\frac{d}{\text{dt}}[\text{mRNA}4] & \;= & \text{cod}4-\text{mRNA}\text{\_}\text{deg}\text{\_}\text{rate}\;[\text{mRNA}4] \end{array}$$

(22) $$\;\begin{array}{cccc} \;\frac{d}{\text{dt}}[p4] & \;= & \;\text{rbs}4\text{\_}\text{strength} & \;[\text{mRNA}4]\\ & & \;-p\text{\_}\text{deg}\text{\_}\text{rate} & \;[p4] \end{array}$$

(23) $$\begin{array}{ccc} \;\frac{d}{\text{dt}}[\text{mRNA}5] & \;= & \text{cod}5-\text{mRNA}\text{\_}\text{deg}\text{\_}\text{rate}\;[\text{mRNA}5] \end{array}$$

(24) $$\;\begin{array}{cccc} \;\frac{d}{\text{dt}}[p5] & \;= & \;\text{rbs}5\text{\_}\text{strength} & \;[\text{mRNA}5]\\ & & \;-p\text{\_}\text{deg}\text{\_}\text{rate} & \;[p5] \end{array}$$

(25) $$\begin{array}{ccc} \;\frac{d}{\text{dt}}[\text{mRNA}6] & \;= & \text{cod}6-\text{mRNA}\text{\_}\text{deg}\text{\_}\text{rate}\;[\text{mRNA}6] \end{array}$$

(26) $$\;\begin{array}{cccc} \;\frac{d}{\text{dt}}[p6] & \;= & \;\text{rbs}6\text{\_}\text{strength} & \;[\text{mRNA}6]\\ & & \;-p\text{\_}\text{deg}\text{\_}\text{rate} & \;[p6] \end{array}$$

where we have used the variables

(27) $$\begin{array}{ccc} \;\text{cod}1 & \;= & \text{pro}1\text{\_}\text{strength} \end{array}$$

(28) $$\begin{array}{ccc} \;\text{cod}2 & \;= & \text{pro}2\text{\_}\text{strength}\;\left(\frac{{\left([p1]/K_{2}\right)}^{h2}}{1+{\left([p1]/K_{2}\right)}^{n2}}\right)\;\\ & & \times \left(\frac{1}{1+{\left([p6]/K_{5}\right)}^{n5}}\right)\; \end{array}$$

(29) $$\begin{array}{ccc} \;\text{cod}3 & \;= & \text{pro}3\text{\_}\text{strength}\;\left(\frac{{\left([p1]/K_{3}\right)}^{h3}}{1+{\left([p1]/K_{3}\right)}^{n3}}\right)\\ & & \times \left(\frac{1}{1+{\left([p2]/K_{4}\right)}^{n4}}\right) \end{array}$$

(30) $$\begin{array}{ccc} \;\text{cod}4 & \;= & \text{pro}4\text{\_}\text{strength}\;\left(\frac{{\left([p1]/K_{1}\right)}^{h1}}{1+{\left([p1]/K_{1}\right)}^{n1}}\right)\\ & & \times \left(\frac{1}{1+{\left([p5]/K_{8}\right)}^{n8}}\right) \end{array}$$

(31) $$\begin{array}{ccc} \;\text{cod}5 & \;= & \text{pro}5\text{\_}\text{strength}\;\left(\frac{1}{1+{\left([p4]/K_{6}\right)}^{n6}}\right) \end{array}$$

(32) $$\begin{array}{ccc} \;\text{cod}6 & \;= & \text{pro}6\text{\_}\text{strength}\;\left(\frac{1}{1+{\left([p4]/K_{7}\right)}^{n7}}\right). \end{array}$$

Notice that the mRNA concentrations each degrade with the same rate *mrna_deg_rate*, which we assume is 1 throughout this paper. Also note that the proteins each decay with the same rate *p_deg_rate*, which is one of the 29 parameters to be inferred. The remaining 28 parameters are the production rates for each component: *rbs[j]_strength* and *pro[j]_strength* for *j* = 1,…,6 and the Michaelis-Menten constants and Hill coefficients: *K*_*j*_ and *h*_*j*_ for *j* = 1,…,8. Measurements are made of the either the mRNA or protein concentrations at specific time points. That is to say, $y_{i}^{\text{obs}}$ in Eq. (3) can correspond to either *mRNA[j]* or *p[j]* at a specific time point.

The perturbation experiments modify the above equations as follows: Deleting a gene corresponds to eliminating production of both the mRNA and protein for the corresponding gene, i.e. *pro[j]_strength* = *rbs[j]_strength* = 0 for the appropriate gene. We implement mRNA knockdown by a five-fold increase in the mRNA degradation rate for the appropriate gene. Increase of protein expression is implemented by doubling the translation rates, *rbs[j]_strength* for *j* = 1,…,6.

## Competing interests

The authors declare that they have no competing interests.

## Author’s contributions

MKT designed the experiment selection method, implemented the numerical simulations, carried out the calculations, and drafted the manuscript. PQ conceived of the study and participated in its design and coordination and helped to draft the manuscript. Both authors read and approved the final manuscript.
